# Long-acting reversible contraception utilization and associated factors among women in extended postpartum period in Hossana town, southern Ethiopia*:* cross sectional study

**DOI:** 10.1186/s40834-020-00117-6

**Published:** 2020-08-05

**Authors:** Biruktawit Fekade Woldu, Tadesse Lelago Ermolo, Lidiya Gutema Lemu, Negeso Gebeyehu Gejo

**Affiliations:** 1 Department of Midwifery, College of Medicine and Health Sciences, Wachemo University, Hossana, Ethiopia; 2 Department of Nursing, College of Medicine and Health Sciences, Wachemo University, Hossana, Ethiopia; 3 Department of Midwifery, College of Health Sciences, Mizan Tepi University, Mizan Aman, Ethiopia

**Keywords:** Long-acting, Contraception, Postpartum, Prevalence, Factors, Hossana

## Abstract

**Background:**

In low and middle-income countries, 95% of postpartum women want to avoid a pregnancy for 2 years, but 70% are not using contraception. Delay in use of contraception by couples during postpartum period can result in many unwanted pregnancies. Long-acting reversible contraception (LARC) is ideal for postpartum women. Therefore this study aimed at assessing the prevalence and factors associated with LARC use among postpartum women.

**Methods:**

Facility based cross sectional study was conducted from July 23-Aug 25, 2018. Systematic random sampling technique was employed to recruit a total of 381 women in extended postpartum period visiting Child Immunization service in hosanna health institutions. Pretested structured questionnaire was used for data collection. Data was analyzed by SPSS version 20. Binary and multiple logistic regression analysis was done. The presence and strength of association was determined using AOR with its 95% CI. Variables with *P* value less than 0.05 were considered as statistical significant.

**Results:**

The prevalence of LARC use was 36.5% (95%CI (33.05–39.95)). The main reason for not using LARC was fear of side effect and false information. Previous use of LARC (AOR = 3.3, 95%CI (1.7–6.5)) and have ever discussed with health providers on LARC (AOR = 2.5, 95%CI (1.1–5.74)) were found to be significantly associated with LARC use.

**Conclusions:**

The utilization of LARC among postpartum women was found to be higher than other studies in Ethiopia. Provision of effective contraceptive counseling during Antenatal, delivery and postnatal care services with emphasis on LARC/Postpartum Intra-Uterine Device is important.

## Background

After one-time placement of a device, Long-acting reversible contraception (LARC) methods provide reliable long-term highly effective prevention of pregnancy. LARC methods include subdermal hormonal implant and Intra-Uterine Devices (hormonal IUDs and non-hormonal copper-containing IUDs) [1]. Regardless of considerable advances in contraceptive technologies, unintended pregnancies remain a significant global public health issue. In developing countries 214 million reproductive age women who desire to avoid pregnancy are not using a modern contraceptive method [2, 3].

Modern contraceptive use has increased around the world. However, less than 15% of married women of reproductive age use a modern method in many of the poorest countries. The use of LARC among modern contraception users is still low [4]. In Ethiopia, Contraceptive prevalence rate among currently married women has increased steadily from 14% in 2005 to 41% in 2019. The largest increase is observed in the use of injectable. The use of LARC remains low at 11% (9%- Implants & 2%-IUD) [5]. In southern region, where the study area is located, 44.6% of currently married women were using a modern family planning. Among these only 9.2% use LARC (7.7%- Implants & 1.5%-IUD). Utilization of LARC was found to have significant association with women’s socio-demographic and reproductive characteristics. Knowledge, attitude and previous LARCs use was also found to have significant association with its use [6–14].

In the first year after childbirth, 95% of women in low and middle-income countries want to avoid pregnancy within the next 2 years; however 70% are not using contraception. Delay in practice of contraception by couples during postpartum period can result in many unwanted or mistimed pregnancies. A woman conceives within/less than 24 months of delivery has higher chances of complications like abortions, pre-term labor, low birth weight babies, postpartum hemorrhage, neonatal morbidity and mortality [15]. Postpartum family planning is defined as the prevention of unintended pregnancy and closely spaced pregnancies through the first 12 months following childbirth [16]. Postpartum period is one of the important and crucial times when women and couples are highly motivated and receptive to family planning methods. LARC methods are safe for almost all women, including those in a postpartum period [2]. As they do not contain estrogen and have few contraindications, they are ideal for use in postpartum women [17]. According to a study in five low-income countries, almost all women wish to delay or prevent a future pregnancy at six weeks postpartum. Despite this, usage of modern contraceptive methods at 42 days post-partum varied widely. The uptake of the most effective LARC (IUDs) is very low even amongst users of modern contraceptives [18]. Different studies conducted in different parts of Ethiopia also indicated lower prevalence of postpartum LARC use [19–24]. Study on LARC use among postpartum women is lacking in the study area. Therefore the objective of this study was to assess the prevalence and factors associated with LARC use among women in extended postpartum period visiting child immunization in Hosanna health institutions, Southern Ethiopia.

## Methods

### Study setting and population

Facility based cross sectional study was conducted from July 23-Aug 25, 2018 in hosanna health institutions, Hadiya zone Southern Ethiopia. Hossana town comprises of three sub cities and eight kebeles(the lowest governmental administrative units). According to CSA population count of May 2007, the total number of Hossana town population was 69,959 for the year 2007/8. Among these 34,454 (49.25%) were females. There is 1 teaching referral hospital and 3 health centers in the town which provide postpartum family planning service. Women in extended postpartum period visiting child immunization service in the four health institutions during the study period were eligible for enrollment. Those women who are pregnant and unable to speak or hear were excluded from the study.

### Sample size determination and sampling technique

Sample size was calculated using single population proportion formula by taking account of Proportion of LARC use among postpartum women in Durame town 36.7% [13], CI = 95% Margin of error = 5 and 10% non-response rate. A total of 392 women on extended postpartum period (6 weeks to 1 year after delivery) were invited to participate. Systematic random sampling technique was employed to select participants. The total number of women who visit child Immunization (N) was estimated by considering the immediate previous 1 month record of Expanded Program on Immunization chart. Then number of sampling intervals (k) was determined by dividing N by sample size (*n* = 392). Then lottery method was used to select the first participant. Then after every 3rd unit was included until the required sample reached. In case of refusal or not meeting inclusion criteria, the next person was selected. The sample size for each institution was distributed proportionally.

### Data collection

Data were collected using pretested, interviewer administered structured questionnaire. The questionnaire was adapted from different literatures by considering the local situation of the study subjects. Information on participants’ socio-demographic, reproductive characteristics, knowledge, attitude and practice towards LARC was collected. The data collection tool prepared in English was translated in to Amharic and then returned back to English to ensure accuracy. Before the actual data collection, Pre-test was done on 5% women in a health center located outside the town and necessary corrections made accordingly. Four trained diploma midwifery graduates collected the data supervised by one BSc midwife working in university. Completeness of the questionnaires was checked on the spot by supervisor.

### Measurement

LARC use: was defined as utilization of any of LARCs (IUD or subdermal hormonal implant) by women on extended postpartum period.

The mean score was used to categorize women’s knowledge towards LARC; women who scored above mean score were considered to have good knowledge and women who scored below the mean were considered to have poor knowledge.

Women’s attitude towards LARC was categorized in the same approach. Those women who scored above the mean were considered to have positive attitude and scored below the mean considered to have negative attitude towards LARC.

### Data analysis

Data was analyzed by using SPSS version 20. Descriptive statistics were computed. Binary logistic regression analysis was done and all explanatory variables with *p* value less than 0.2 were entered to multiple logistic regression model. The second analytical step used a stepwise backward model. Then association was assessed using AOR and its 95%CI. And *p* value of < 0.05 was considered as statistically significant. Goodness of model fitness was tested by using Hosmer and Lemeshow test.

## Results

Data were analyzed from 381 women, making response rate of 97.1%. Mean age of participants were 27(SD ± 4.6). Majority of participants were urban resident (89%). Less than one fifth of respondents attend diploma and above level of education (17.1%). Half of the women were housewife (50.4%) (Table 1).

**Table 1:** Sociodemographic characteristics of postpartum women in Hossana health institutions, 2018(*n* = 381)

Variable	Frequency	Percentage
Age (years)		
16–20	31	8.1
21–25	124	32.5
26–30	154	40.4
31–35	50	13.1
36–40	22	5.8
Residence		
Urban	339	89.0
Rural	42	11.0
Marital status		
Married	369	96.9
Single	7	1.8
Separated	5	1.3
Educational status		
Can’t read and write	15	3.9
Can Read & Write	19	5.0
Primary	141	37.0
Secondary	141	37.0
Diploma & Above	65	17.1
Working status		
Housewife	192	50.4
Merchant	51	13.4
Government Employee	67	17.6
Private Employee	58	15.2
Other	13	3.4

### Reproductive characteristics of respondents

More than two-third (71%) of participants had two or more births. Median number of alive children per women and birth interval were 2 IQR [1, 4] and 36 months IQR [24,48] respectively. Around one-third (28.3%) participants do not want any more children for the future.

Less than one fifth of respondents (18.1%) intended to give birth within 2 years. More than one-forth (28.6%) had never discussed with health professionals towards LARC and 52.4% were advised on LARC during the immediate postpartum period (Table 2).

**Table 2:** Reproductive characteristics of postpartum women in Hossana health institutions, 2018(*n* = 381)

Variable	LARC use	
	Yes n(%)	No n(%)
Parity		
1	72(65.5)	38(34.5)
2–4	137(61.7)	85(38.3)
≥ 5	33(67.3)	16(32.7)
Number of alive children		
1	75(65.8)	39(34.2)
2–4	135(60.8)	87(39.2)
≥ 5	32(71.1)	13(28.9)
Place of last birth		
Home	15(71.4)	6(28.6)
Health institution	227(63.1)	133(36.9)
Birth interval(in month) (*n* = 272)		
0–23	28(73.7)	10(26.3)
24–35	56(64.4)	31(35.6)
36 & above	87(59.2)	60(40.8)
Number of children want for the future		
0	74(68.5)	34(31.5)
1–3	131(59.8)	88(40.2)
4–6	37(68.5)	17(31.5)
Intended to give birth within 2 years (*n* = 273)		
Yes	49(71.0)	20(29.0)
No	119(58.3)	85(41.7)
Ever discussed with health professionals		
Yes	123(45.2)	149(54.8)
No	16(14.7)	93(85.3)
Advised during immediate postpartum		
Yes	95(47.5)	105(52.5)
No	44(24.3)	137(75.7)

### Knowledge, attitude and previous LARC use

More than two-third (71.4%) of the women had good knowledge towards LARC. Around half of them (53.2%) had positive attitude towards LARC. Two-third (68.5%) of participant’s partner had supportive attitude towards LARC. Two-third (66.4%) of respondents had ever used at least one type of modern contraception and one-fourth (26%) had ever used LARC before delivery of last child (Table 3).

**Table 3:** Knowledge, attitude and previous LARC use among postpartum women in Hossana health institutions, 2018(*n* = 381)

Variable	LARC use	
	Yes	No
Knowledge towards LARC		
Good	121(44.5%)	151(55.5%)
Poor	18(16.5%)	91(83.5%)
Attitude towards LARC		
Positve	77(41.8%)	107(58.2%)
Negative	61(38.4%)	98(61.6%)
Previous LARC use		
Used before	58(59.2%)	40(40.8%)
Never used	81(28.6%)	202(71.4%)

### Prevalence of postpartum LARC use

Two-third (66.6%) of postpartum women had started to use modern family planning method. LARC method use among participants was 36.5% (implant 34.1%, IUCD-2.4%). The main reason for not using LARC was fear of side effect (39.3%) followed by false rumors towards LARC (it cause harm to uterus, infertility, interfere with work, fly/lost in the body) (21.9%) (Table 4).

**Table 4:** method-mix adopted among postpartum women in Hossana health institutions, 2018(*n* = 381)

Contraception method	Frequency	Percentage
Injectable	98	25.7
Implants	130	34.1
IUCD	9	2.4
Pills	15	3.9
Condom	2	0.5
Non users	127	33.4

### Factors associated with LARC use

In the multiple logistic regression analysis Previous LARC use and ever discussed with Health Professionals on LARC were found to be significantly associated with LARC use. Those women who have ever used LARC before were 3.3 times more likely to use LARC on postpartum period (AOR = 3.3, 95%CI(1.7–6.5). Postpartum women who have ever discussed with health professionals on LARC were 2.5 times more likely to use LARC (AOR = 2.5, 95% CI (1.1–5.7)) (Table 5).

**Table 5:** Logistic regression analysis of factors associated with LARC use among postpartum women in hossana health institutions, 2018 (*n* = 381)

Variable	LARC use		COR (95%CI)	AOR (95%CI)
	Yes (n%)	No (n%)		
Residence				
Urban	130(38.3)	209(61.7)	1	
Rural	9(21.4)	33(78.6)	0.43 (.20–.94)	0.53 (0.19–1.42)
Intention to give birth within 2 years				
Yes	20(29.0)	49(71.0)	1	
No	85(41.7)	119(58.3)	1.75(.97–3.15)	1.82 (0.94–3.54)
Knowledge towards LARC				
Good	121(44.5)	151(55.5)	4.01(2.31–7.08)	0.95 (0.43–2.10)
Poor	18(16.5)	91(83.5)	1	
Partner attitude				
Supportive	118(45.2)	143(54.8)	2.59 (1.48–4.54)	0.50 (0.24–1.03)
Not supportive	20(24.1)	63(75.9)	1	
Previous LARC use				
Yes	58(59.2)	40(40.8)	3.61 (2.24–5.83)	3.34(1.70–6.55)*
No	81(28.6)	202(71.4)	1	
Adviced during immediate postpartum				
Yes	95(47.5)	105(52.5)	2.81 (1.81–4.36)	1.26 (0.66–2.42)
No	44(24.3)	137(75.7)	1	
Ever discussed on LARC with professionals				
Yes	123(45.2)	149(54.8)	4.79 (2.68–8.58)	2.56 (1.14–5.74)**
No	16(14.7)	93(85.3)	1	

*Significant at < 0.05 ** significant at < 0.001

## Discussion

In this study the prevalence of LARC use among postpartum women was found to be 36.5% (95%CI (33.05–39.95)), which is similar with a study done in Durame [13]. However it is higher than a study in Northern Ethiopia [24]. The difference can be due to variation in participants’ characteristics and institution based nature of our study.

Majority of LARC users opted for Implant (93.5%) and very few used IUCD (6.5%). Which is consistent with different studies [6, 14] where strong preference for implants over IUCD were observed. These can be due to prevailing misinformation towards IUCD.

The main reason for not using LARC among postpartum women was fear of side effect (39.3%) followed by false information towards LARC (it cause harm to uterus and infertility, interfere work, fly/lost in the body) (21.9%) which is consistent with other study [8]. This indicates the need of community based health education and awareness creation programs.

In this study previous LARC use was found to be significantly associated with postpartum LARC use (AOR = 3.34 (1.70–6.55)). Similar finding were indicated by other studies [10, 13]. The possible explanation can be women possibly will have adequate information on the contraception method they used before, which can motivate them to use the same method repetitively.

Those postpartum women who ever discussed with health professionals on LARC were found to be more likely to use LARC (AOR = 2.569(1.148–5.7470)). It is in line with another study [6]. This can possibly explained by the fact that access to clear factual information on the benefits and limitations of contraceptive methods is imperative to promote informed choice and to overcome misperceptions and myths more broadly perpetuated in the community.

### Limitation

Quantitative nature of the study hinders indepth exploration of women’s perception and barrieres for LARC use. As this study was conducted in urban facilities, the finding might not be representative for the rural communities.

## Conclusion

The utilization of LARC among postpartum women was found to be higher than other studies done in Ethiopia. Majority of participants opted for implants over IUCD. The main reason for not using LARC was fear of side effect and rumors. Provision of effective contraceptive counseling during Antenatal, delivery and postnatal care services with emphasis on LARC/ PPIUDs is important. Further studies need to be conducted to assess women’s knowledge, perceptions and attitudes towards PPIUDs.

## Data Availability

The datasets used and/or analysed during the current study are available from the corresponding author on reasonable request.
